# Olig2-astrocytes express neutral amino acid transporter SLC7A10 (Asc-1) in the adult brain

**DOI:** 10.1186/s13041-021-00874-8

**Published:** 2021-11-08

**Authors:** Kouko Tatsumi, Kaoru Kinugawa, Ayami Isonishi, Masahiro Kitabatake, Hiroaki Okuda, Shoko Takemura, Tatsuhide Tanaka, Eiichiro Mori, Akio Wanaka

**Affiliations:** 1 Department of Anatomy and Neuroscience, Faculty of Medicine, Nara Medical University, Kashihara, Nara 634-8521 Japan; 2 Department of Neurology, Faculty of Medicine, Nara Medical University, Kashihara, Nara 634-8521 Japan; 3 Department of Future Basic Medicine, Faculty of Medicine, Nara Medical University, Kashihara, Nara 634-8521 Japan; 4 Department of Immunology, Faculty of Medicine, Nara Medical University, Kashihara, Nara 634-8521 Japan; 5 Department of Anatomy, Graduate School of Medical Science, Kanazawa University, Kanazawa, Ishikawa 920-1192 Japan

## Abstract

We have reported that the transcription factor Olig2 labels a subpopulation of astrocytes (Olig2-astrocytes), which show distribution patterns different from those of GFAP-expressing astrocytes (GFAP-astrocytes) in the adult brain. Here, to uncover the specific functions of Olig2-astrocytes, we first analyzed public single-cell RNA-seq databases of adult mouse brains. Unbiased classification of gene expression profiles and subsequent gene ontology analyses revealed that the majority of Olig2-astrocytes belonged to an astrocytic cluster that is enriched for transporter-related genes. SLC7A10 (also known as ASC-1) was one of the representative neutral amino acid transporter genes in the cluster. To complement the in silico data analyses, we differentially isolated Olig2- and GFAP-astrocytes from the same frozen section of the lateral globus pallidus using laser microdissection and compared their gene expression by quantitative reverse transcription PCR. We confirmed that Olig2 and GFAP mRNAs were preferentially expressed in the Olig2- and GFAP-astrocytes, respectively, indicating that the laser microdissection method yielded minimal cross-contamination between two types of cells. The Olig2-astrocytes expressed significantly higher levels of SLC7A10 mRNA than the GFAP-astrocytes, corroborating the in silico data. We next localized SLC7A10 protein by immunohistochemistry in the lateral globus pallidus, which was also genetically labeled for Olig2. SLC7A10 co-localized with Olig2-genetic labeling, especially on the fine processes of Olig2-astrocytes. These results are consistent with the recent discovery that SLC7A10 is expressed not only in neurons but also in a subset of astrocytes. Taken together, our findings suggest that SLC7A10 exerts specific functions in Olig2-astrocytes of the adult brain.

**Supplementary Information:**

The online version contains supplementary material available at 10.1186/s13041-021-00874-8.

## Introduction

The Olig2 gene controls the development and differentiation of oligodendrocyte lineage cells in the central nervous system [1, 2]. Recently, we and other groups have found that Olig2 also labels astrocytes in the adult mouse brain [3–8]. During our genetic labeling studies using transgenic mice [3, 9–11], we noticed that Olig2-positive cells constituted a subpopulation of astrocytes in the adult brain, both under physiological conditions and after injury. Notably, Olig2-astrocytes expressed a very low level of GFAP protein, an established marker of astrocytes, and were distributed in a pattern different from that of GFAP-astrocytes [3]. Even within a single brain nucleus, such as the lateral globus pallidus (LGP), Olig2- and GFAP-astrocytes occupied mutually exclusive territories [3, 4]. Reinforcing our findings, an in vitro study recently demonstrated that human iPS cell-derived astrocytes comprise two functionally different populations: one, positive for Olig2 gene expression, was neuroprotective while the other, without Olig2 expression, was not [6]. The Olig2 gene thus seems to play a role in generating astrocytic heterogeneity, and the regional heterogeneity of Olig2-astrocyte distribution implies that each subpopulation has specific and different functions.

Here, to interrogate the specific functions of the Olig2-astrocytes, we compared the gene expression profiles of Olig2- and GFAP-astrocytes by two methods: first, in silico analysis using published single-cell RNA-sequence datasets containing transcriptomes of whole mature mouse brains [12]; and second, mRNA expression analysis using differentially isolated Olig2- and GFAP-astrocytes from a single section of the LGP by laser microdissection in combination with immunohistochemistry methods. The reason we focused on the LGP is that Olig2- and GFAP-astrocytes intermingled and occupied mutually exclusive territories in this nucleus, with possible contrast in function and gene expression. The microdissected samples were subjected to quantitative RT-PCR analyses after mRNA extraction.

We identified differentially expressed genes in the Olig2- and GFAP-astrocytes. Among them, mRNA for SLC7A10, a neutral amino acid transporter, was preferentially expressed in the Olig2-astrocytes. SLC7A10 protein was also immunolabeled in the Olig2-astrocytes of the LGP, especially in their fine processes.

## Results

### Single-cell RNA sequencing

**Fig. 1:**
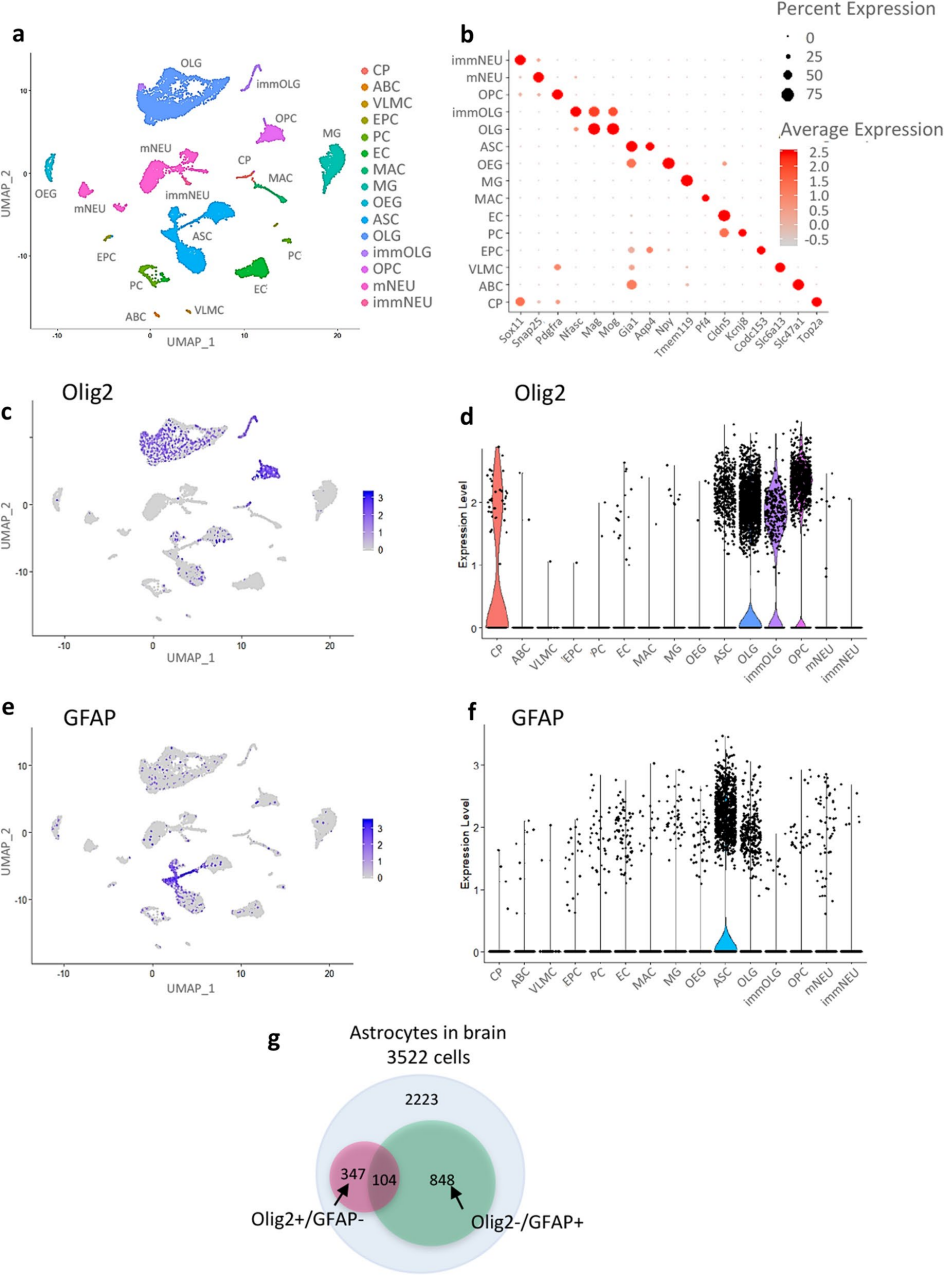
Identification of cell types from whole-brain cells. **a** UMAP plot shows the unsupervised clustering of 16,028 cells derived from mouse brain (for details, see “Methods”). The 15 cell types were classified by their transcriptional profiles. **b** Dot plot shows the expression levels of a representative cell-specific marker gene for each cell type. **c** UMAP plot shows the distribution of Olig2 expression. **d** Violin plot shows Olig2 expression across all clusters. **e** UMAP plot shows the distribution of GFAP expression. **f** Violin plot shows GFAP expression across all clusters. **g** A total of 3522 cells of the astrocyte subset were derived from cells in mouse brain. They were classified into Olig2(+)/GFAP(−) astrocytes (347 cells), Olig2(+)/GFAP(+) astrocytes (104 cells), Olig2(−)/GFAP(+) astrocytes (848 cells) and Olig2(−)/GFAP(−) astrocytes (2,223 cells). *immNEU* immature neuron, *mNEU* mature neuron, *OPC* oligodendrocyte precursor cell, *immOLG* immature oligodendrocyte, *OLG* oligodendrocyte, *ASC* astrocyte, *OEG* olfactory ensheathing glia, *MG* microglia; *MAC* macrophage, *EC* endothelial cell, *PC* pericyte, *EPC* ependymal cell, *VLMC* vascular and leptomeningeal cell, *ABC* arachnoid barrier cell, *CP* cycling progenitor

**Fig. 2:**
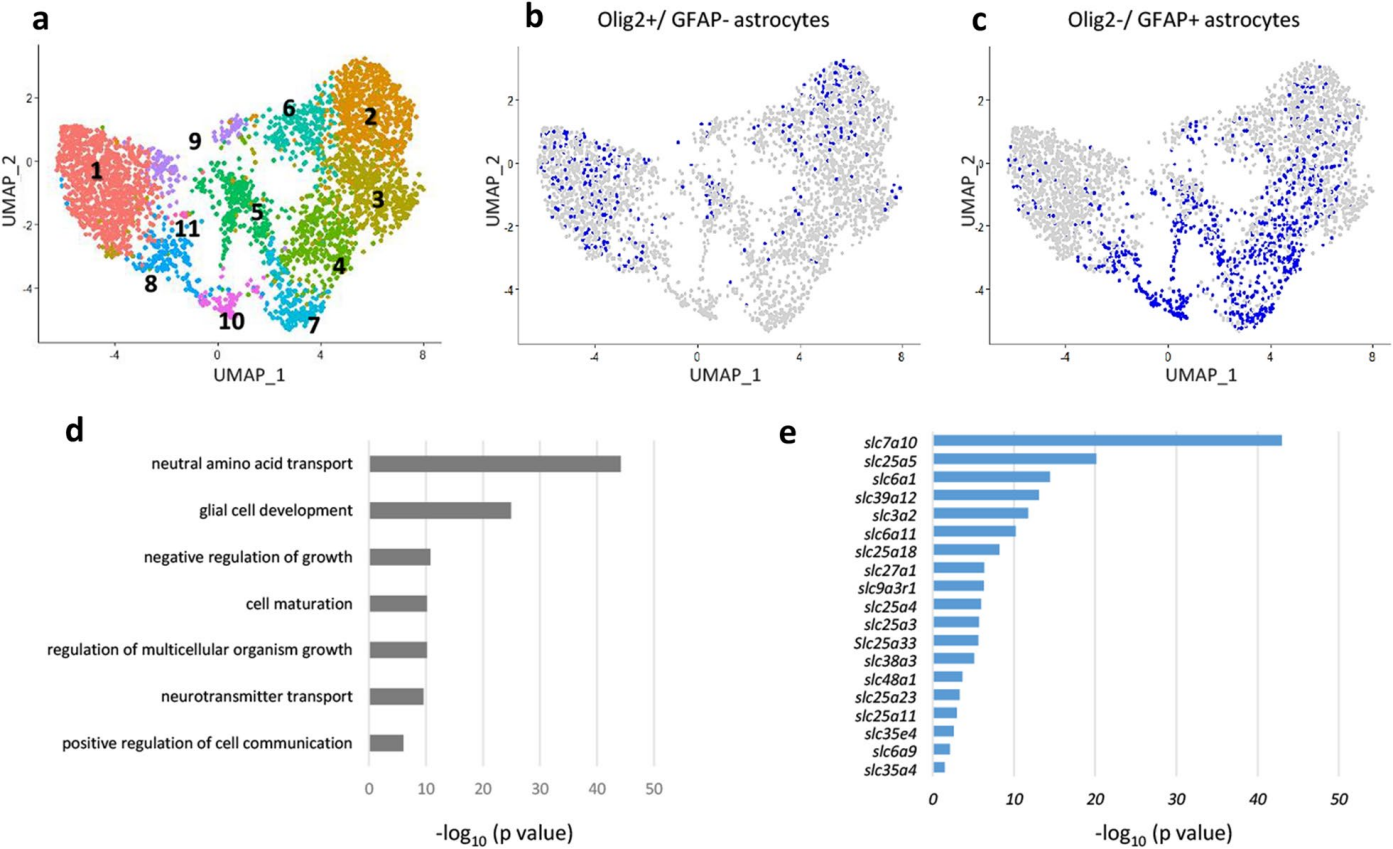
Identification of astrocytic clusters from whole-brain cells. **a** UMAP plot classifies the 3522 astrocytes into eleven clusters (A1–A11) by unsupervised re-clustering. **b**, **c** Distribution of Olig2(+)/GFAP(−) astrocytes (347 cells) (**b**) and Olig2(−)/GFAP(+) astrocytes (848 cells) (**c**) on UMAP plots. **d** Ontology (GO) analysis on the group A1 gene set. The list shows the top seven GO terms obtained ranked by p value (p < 0.05, adjusted p value based on Bonferroni correction). **e** SLC genes extracted from 416 genes whose expression was significantly biased to Olig2-astrocytes as compared to GFAP-astrocytes (p < 0.05, adjusted p value based on Bonferroni correction)

We analyzed an scRNA-seq dataset that was previously published [12] (see “Methods”). This dataset contains transcriptomes derived from mature mouse brains. After quality control and filtering, we analyzed a total of 16,028 single cells with 14,658 genes from the brains of eight young (2–3 months old) mice. We performed a principal component analysis (PCA), unsupervised clustering, and dimensionality reduction with Uniform Manifold Approximation and Projection (UMAP). In our initial analysis, we obtained 28 clusters and categorized them into 15 clusters of the major cell types (Fig. 1a) based on their gene expression profiles. Representative cell-specific marker genes were expressed in the corresponding cluster (Additional file 1: Fig. S1). A dot plot showed the expression levels of marker genes for each cell type (Fig. 1b). For example, the mature neuron (mNEU) cluster expressed synaptosome-associated protein 25 (Snap25), which is a pan-neural marker. Similarly, the oligodendrocyte (OLG) cluster expressed myelin-associated glycoprotein (Mag) and myelin oligodendrocyte glycoprotein (Mog), and the astrocyte (AS) cluster expressed aquaporin 4 (AQP4) and gap junction protein alpha 1 (Gja1). The UMAP plot showed that a total of 16,028 single cells were successfully classified into cell-type clusters. We then focused on Olig2 gene-expressing cells (a total of 3626 cells). Consistent with our knowledge and previous literature [1, 10, 13], they were mainly allocated to the oligodendrocyte lineage clusters containing immature oligodendrocytes (immOLGs), oligodendrocyte precursor cells (OPCs) and OLGs (3091 cells) (Fig. 1c). In addition, Olig2-expressing cells were found in the AS cluster (451 cells) (Fig. 1c). A violin plot underlined the above finding: Olig2-expressing cells accumulated in oligodendrocyte lineage clusters and in the AS cluster (Fig. 1d). NEU lineage (mature and immature neurons), MG (microglia) and other clusters showed few Olig2-expressing cells. By contrast, most of the GFAP gene-expressing cells accumulated in the AS cluster (952 cells in a total of 1612 cells) (Fig. 1e). AS was the dominant cluster for GFAP-expressing cells, followed by OLG and mNEU and other clusters (Fig. 1f). Taken together, these unbiased scRNA-seq analyses confirmed that Olig2 expression occurred not only in oligodendrocyte lineage cells but also in astrocytes of the adult brain, corroborating our previous report [3]. We classified the AS cluster into four subsets according to the expression patterns of two genes, Olig2 and GFAP: Olig2(+)/GFAP(−) astrocytes (347 cells), Olig2(+)/GFAP(+) astrocytes (104 cells), Olig2(−)/GFAP(+) astrocytes (848 cells) and Olig2(−)/GFAP(−) astrocytes (2223 cells) (see “Methods”; Fig. 1g). Next, to further characterize molecular diversity in astrocytic populations, we performed additional analysis on the AS cluster (a total of 3522 cells with 14,658 genes; see “Methods”). This second analysis successfully sorted 11 groups (hereafter referred to as A1–A11) based on enriched gene expression profiles (Fig. 2a, Additional file 3: Table S2). We allocated Olig2+/GFAP- astrocytes (347 cells) and Olig2-/GFAP+ astrocytes (848 cells) to the 11 groups (Fig. 2b, c, respectively). As expected, the two astrocytic subpopulations showed different distribution patterns. Because Olig2-astrocytes tended to accumulate in group A1 (47%, 163/347 cells) (Fig. 2b, Additional file 4: Table S3), we examined the biological features by Gene Ontology (GO) analysis of the group A1 gene set. This showed that group A1 is enriched for neutral amino acid transporter genes (GO: 0015804) (Fig. 2d) including solute carrier family 6 member 9 (SLC6A9, also known as GLYT1), SLC3A2 (4F2 Heavy Chain) and SLC7A10 (ASC-1) (Additional file 5: Table S4). We further extracted 416 genes whose expression was significantly biased to Olig2-astrocytes as compared to GFAP-astrocytes (p < 0.05, adjusted p value based on Bonferroni correction) (Additional file 6: Table S5). Among 19 SLC genes extracted from the 416 genes, SLC7A10 showed the highest level of gene expression (Fig. 2e, Additional file 7: Table S6). Based on these in silico data, we focused on the SLC7A10 gene, and subjected it to RNA expression analyses and immunohistochemistry (see below).

### Differential isolation of Olig2- and GFAP-astrocytes from the LGP

**Fig. 3:**
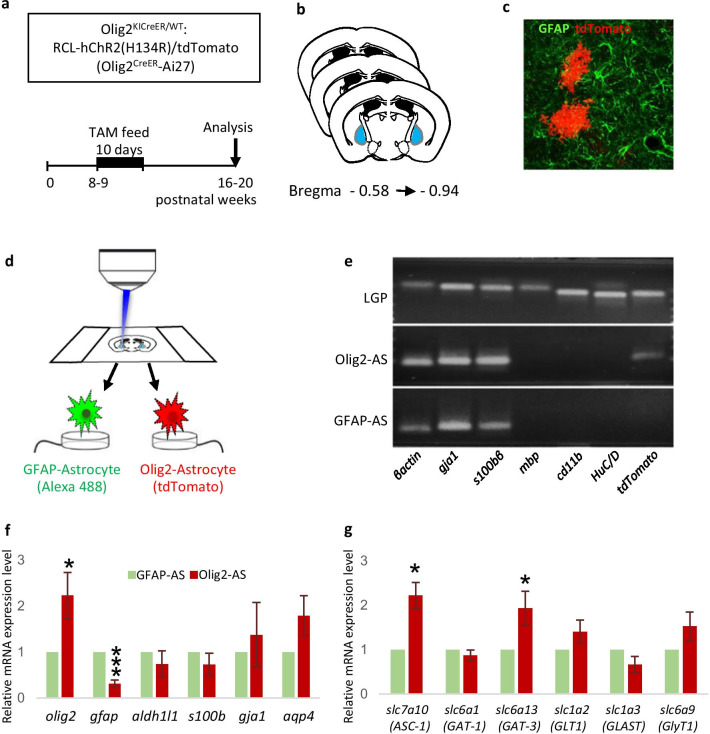
Olig2- and GFAP-astrocytes can be differentially collected from the LGP in Olig2^CreER^:ROSA-tdTomato mice by laser microdissection. **a** Experimental schedule of tamoxifen (TAM) administration. **b** Histological localization of the LGP of the mouse [40]. Numerical values indicate posterior distance (mm) from the plate of Bregma. **c** Olig2-astrocytes (tdTomato red fluorescence) and GFAP-immunolabeled astrocytes (green fluorescence) show mutually exclusive localization in the LGP. **d** Schematic representation of the selective dissection of Olig2- and GFAP-astrocytes by laser microdissection. **e** PCR analysis of selected transcripts in laser-microdissected LGP (top), Olig2-astrocytes (middle) and GFAP-astrocytes (bottom). There is no mutual contamination in the collected samples between Olig2- and GFAP-astrocytes and other cell types. These samples derive from the same experiment and the gels were processed in parallel. Full-length gels are presented in Additional file 1: Fig. S1. **f** RT-qPCR analysis for mRNA expression levels of astrocyte-specific genes in the two distinct populations of astrocytes. *gfap* mRNA expression in Olig2-astrocytes was significantly lower than that in GFAP-astrocytes, and there was no significant difference for other pan-astrocytic marker genes. **g** RT-qPCR analysis for mRNA expression levels of SLC membrane transporter genes. *slc7a10* and *slc6a13* mRNA expression was significantly higher in Olig2-astrocytes than in GFAP-astrocytes. Graphical data are presented as the mean ± SEM. Student’s t-test was used to compare mean values for unpaired data. Differences were considered significant when **p < 0.01, ***p < 0.001

Next, we isolated Olig2- and GFAP-astrocytes from the LGP*.* Since the LGP is a representative nucleus where many Olig2- and GFAP-astrocytes intermingle and occupy mutually exclusive territories [3], it is of interest to know how different the gene profiles of two adjacent populations are. We first double-stained the LGP section with anti-GFAP antibody and with genetic Olig2-labeling [3]. For the latter labeling, we limited the period of oral tamoxifen administration to 10 days (for maximum recombination we usually administer it for 5 weeks) (Fig. 3a) and localized Olig2-astrocytes in coronal sections containing the LGP (Fig. 3b). Continuous feeding with tamoxifen-containing chow markedly induced Cre-mediated recombination, yielding robust tdTomato fluorescence labeling of Olig2-lineage cells. As demonstrated in our previous study [3], Olig2-astrocytes (tdTomato-positive) alternated with GFAP-astrocytes (Alexa 488-labeled) in the LGP (Fig. 3c), and we could differentially isolate them from a section (Fig. 3d). Although we occasionally observed cells labeled with both markers, we could easily identify these by yellow fluorescence and thus omit them from the cellular collection. We also took care in laser microdissection to avoid cellular border regions where the two populations interdigitate with each other. With these precautions, we could minimize mutual contamination between Olig2- and GFAP-astrocytes as well as contamination with other cell types such as neurons, microglia and oligodendrocytes. To confirm that there was little contamination in our laser-microdissected samples, we performed PCR analyses of cellular markers (Fig. 3e). In the Olig2-astrocyte sample, *gja1* and *s100β* bands (both are astrocytic markers) were detected, but *mbp* (oligodendrocyte), *cd11b* (microglia), and *HuC/D* (neuron) were not amplified (Fig. 3e). For the GFAP-astrocytes, we observed the same results as those for Olig2-astrocytes (Fig. 3e). It should be noted that the *tdTomato* band was detected only for Olig2-astrocytes, indicating that mutual contamination was at a negligible level. Quantitative PCR analysis showed that there was no significant difference in the expression of the pan-astrocytic marker genes *aldh1l1* (0.74 ± 0.29, p = 0.23, n = 3), *s100β* (0.73 ± 0.24, p = 0.14, n = 5), *gja1* (1.4 ± 0.7, p = 0.32, n = 3) and *aqp4* (1.8 ± 0.44, p = 0.08, n = 4) between Olig2- and GFAP-astrocytes (Fig. 3f). Consistent with our previous study [3], *gfap* mRNA expression was significantly lower in Olig2-astrocytes than in GFAP-astrocytes (0.31 ± 0.09, p = 0.0002, n = 6) (Fig. 3f). These data indicated that we successfully isolated Olig2- and GFAP-astrocytes without contamination*.*

### Isolated Olig2-astrocytes in the LGP exhibit significantly higher expression of *slc7a10*.

To confirm the results of in silico analysis, we performed RT-qPCR using isolated astrocytes from the LGP. In addition to *slc7a10*, five astrocytic SLC membrane transporters were tested: *slc6a1* (also known as GAT-1) and *slc6a13* (GAT-3) for transporting GABA, *slc1a2* (GLT-1) and *slc1a3* (GLAST) for transporting glutamate, and *slc6a9* (GlyT1) for glycine transport. The expression levels of *slc7a10* and *slc6a13* were significantly higher in Olig2-astrocytes (*slc7a10*: 2.22 ± 0.29, p = 0.013, n = 4; *slc6a13*: 1.94 ± 0.38, p = 0.02, n = 8). On the other hand, *slc1a, slc1a3* and *slc6a9* did not show a significant difference between the two populations (*slc1a2*: 1.41 ± 0.26, p = 0.13, n = 5; *slc1a3*: 0.66 ± 0.18, p = 0.11, n = 3; *slc6a9:* 1.53 ± 0.33 p = 0.10, n = 4) (Fig. 3g). Expression of another GABA transporter, *slc6a1*, also showed no significant difference (0.87 ± 0.12, p = 0.11, n = 4), perhaps because it is mainly expressed in neurons, and not in astrocytes, in the LGP [14]. These results matched the in silico findings that Olig2-astrocytes expressed significantly more *slc7a10* than GFAP-astrocytes, and also supported our previous hypothesis that Olig2-astrocytes are associated with inhibitory terminals in the LGP via the *slc6a13* transporter [3].

### Immunohistochemical detection of SLC7A10 expression in Olig2-astrocytes

**Fig. 4:**
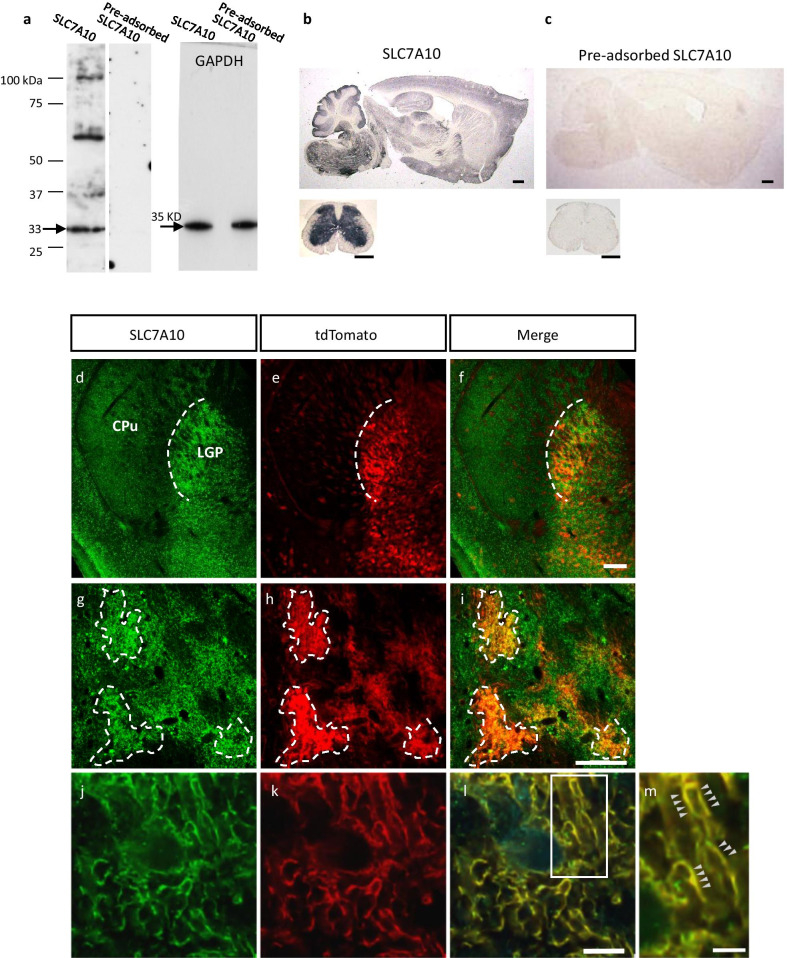
SLC7A10 immunoreactivity is detected in Olig2-astrocytes. **a** The specificity of the primary antibody was confirmed by pre-adsorption assay. Protein from mouse brainstem (15 μg) was separated on by SDS-PAGE and reacted with anti-SLC7A10, pre-adsorbed anti-SLC7A10 and anti-GAPDH antibody. Pre-adsorption was performed with excess SLC7A10 recombinant peptide. The 33-kDa band labeled with SLC7A10 antibody (arrow) was eliminated by incubation with pre-adsorbed antibody. **b** Immunohistochemistry was performed on the mouse brain and spinal cord sections. Immunoreactivity of SLC7A10 was detected in the caudal brain, brainstem and gray matter of the spinal cord. **c** Immunoreactivity of SLC7A10 was completely eliminated in the staining with pre-adsorbed antibody. **d**–**f** Immunofluorescence staining was performed on brain sections of Olig2-Ai27 mice. Strong SLC7A10 immunoreactivity (**d**) was observed in the LGP where tdTomato-labeled Olig2-lineage cells (**e**) were localized (**f** merged image). The border between the caudo-putamen (CPu) and the LGP is marked with a dashed line. **g**–**i** SLC7A10-immunoreactive cells in the LGP. The immunoreactive cells have bushy morphology (**g** demarcated with dashed lines) and show overlapping distribution with Olig2-astrocytes labeled with tdTomato (**h**). Merged image (**i**). **j**–**m** 3D-deconvolution of stacked images of astrocytic processes. SLC7A10 immunoreactivity (**j**) was colocalized on Olig2-astrocytic processes labeled with tdTomato (**k**). Merged image (**l**). **m** Enlarged image of the boxed region in **l**. Note the clear co-localization of SLC7A10 immunoreactivity and tdTomato fused with channelrhodopsin (H134R) on the plasma membrane (arrowheads). Scale bars: **f** 500 μm also for **d** and **e**; **i** 50 μm also for **g** and **h**; **l** 5 μm also for **j** and **k**; **m** 2 μm

Finally, we used immunohistochemistry to examine whether Olig2-astrocytes express SLC7A10, with a specific anti-SLC7A10 antibody. The specificity of the antibody was verified by the absence of staining with antibody pre-absorbed with antigen (Fig. 4a–c). Consistent with previous reports [15, 16], the prominent SLC7A10 immunoreactivity (SLC7A10-ir) was enriched in the caudal forebrain, brain stem and spinal cord (Fig. 4b). We observed strong immunoreactivity in the LGP (Fig. 4d), and the localization pattern was similar to that of Olig2-astrocytes genetically labeled with tdTomato (Fig. 4e, f). Although SLC7A10-ir showed a diffuse staining pattern (Fig. 4b), we found that SLC7A10-ir displayed bushy morphology in the LGP (Fig. 4g) and partially co-localized with tdTomato (Fig. 4h, i). The 3D deconvolution imaging revealed that SLC7A10-ir co-localized with Olig2-astrocytes expressing cytoplasmic tdTomato (Fig. 4j–l). Fine processes of the Olig2-astrocytes were double-labeled for SLC7A10 and tdTomato (Fig. 4m). These findings are consistent with a recent report that SLC7A10 is expressed in a subset of astrocytes [15]. The present study thus demonstrates that Olig2-astrocytes are a source of astrocytic SLC7A10 not only in the spinal cord but also in the forebrain.

## Discussion

Accumulating evidence has revealed that astrocytes display regional and functional heterogeneity in the healthy and unhealthy mouse brain [17–19]. Here, we aimed to interrogate gene expression profiles of Olig2-astrocytes, which we previously identified as a subpopulation of astrocytes in the adult mouse brain [3]. Gene(s) expressed specifically in Olig2-astrocytes, but not in GFAP-astrocytes, may provide clues to the unique functions of Olig2-astrocytes.

Single-cell RNA sequencing (scRNA-seq) is an emerging technique that can analyze transcriptomes at the single-cell level with the aid of powerful next-generation DNA sequencers [20–24]. Based on the expression of thousands of genes in single cells, scRNA-seq analysis has allowed us to identify homologous cell types, gene regulatory networks and molecular heterogeneity among cells [25]. Recently, a database of the transcriptomes of mouse single cells has been published [26]. This database contains gene expression data from approximately 100,000 cells from 20 organs and tissues. In addition, Ximerakis et al. performed scRNA-seq analysis of the brains of eight mature mice (2–3 months) and eight aged mice (21–23 months) using 24,401 and 25,811 cells, respectively, and made the data available for public use [12]. These scRNA-seq databases have paved an unprecedented path to investigate gene regulation in cell populations whose characteristics were unknown. In the present study, we extracted and visualized astrocytic subpopulations from the scRNA-seq database of the brains of eight mature mice [12]. The unbiased classifications clarified the molecular signature of the Olig2-astrocytes. GO analysis indicated that Olig2-astrocytes were enriched for transporter-related genes, especially neutral amino acid transporters. We noticed that SLC3A2, which is inactive as a monomer but functional as a heterodimer with SLC7A10 [27], was also extracted among Olig2-astrocyte-enriched genes. High expression of SLC3A2 underlines the functional importance of SLC7A10 and led us to focus on the latter protein in subsequent analyses. One of the advantages of scRNA-seq analyses is to overview large numbers of genes and we are interested in the 416 genes whose expression is biased to the Olig2(+)/GFAP(−) astrocytes. With different criteria and from different viewpoints, we should be able to identify pivotal genes working in Olig2-astrocytes from these 416 candidates (Additional file 6: Table S5).

To complement the scRNA-seq data with RT-qPCR data, we needed to purify subsets of astrocytes from the adult mouse brain. The FACS method has been employed to obtain specific types of astrocytes [18, 19, 28]. FACS is a useful tool to isolate and to enrich specific cell populations in sufficient quantities for comprehensive gene or proteomic analyses, but it inherently lacks positional (e.g., brain regions and cell layers) and morphological (e.g., cells with or without many processes) information about the isolated cells. We also found non-negligible mutual contamination of the two populations in our preliminary experiments. Laser microdissection can cover the above-listed shortcomings of FACS and can collect cells with defined morphologies under microscopy in certain brain regions [29–31]. In the present study, morphological information was very important to correctly identify Olig2-astrocytes because tdTomato-positive cells contain oligodendrocyte lineage cells in addition to the Olig2-astrocytes in the adult brain [9–11]. Accordingly, laser microdissection in combination with genetic labeling and immunohistochemistry is suited for selectively dissecting Olig2-astrocytes (tdTomato; red fluorescence) and GFAP-astrocytes (Alexa 488; green fluorescence) from a single section. Subsequent PCR analysis confirmed that purified samples had little contamination of oligodendrocyte (MBP), microglia (CD11b) or neuron (HuC/D) markers. Importantly, tdTomato was detected only for the Olig2-astrocytes, and not for the GFAP-astrocytes (Fig. 3e). In agreement with our previous report [3], RT-qPCR analyses showed that Olig2-astrocytes expressed GFAP mRNA weakly (Fig. 3f). These results indicated that we successfully isolated Olig2- and GFAP-astrocytes in the LGP, with a negligible amount of contamination. To our knowledge, this is the first report of two types of cells in a single brain nucleus being differentially isolated by laser microdissection and by different cell markers. In agreement with the in silico data, *slc7a10* showed significantly higher expression in Olig2-astrocytes than in GFAP-astrocytes (Fig. 3g). Interestingly, we also confirmed the higher expression of *slc6a13* (GAT-3), although we did not find GAT-3 among the genes enriched in Olig2-astrocytes in silico (Additional file 6: Table S5)*.* This discrepancy likely comes from the method of analyzing the scRNA-seq database: the SLC6A13 gene is classified as a neurotransmitter, and is not included in the gene sets used for clustering the astrocytic group.

SLC7A10 was originally known as sodium-independent alanine–serine–cysteine transporter-1. Previous reports have demonstrated that SLC7A10 has a high affinity for d-serine and glycine in association with SLC3A2 (also known as 4F2 heavy chain) [16, 27] and it was thought to work mainly in neurons. But a recent study using *Slc7a10*^*tm1Dgen*^ mice revealed that SLC7A10 was substantially enriched in a subset of astrocytes but not in neurons [15]. The astrocytic SLC7A10 expression was dominant in the caudal forebrain, the brain stem and the spinal cord, where glycinergic inhibitory synapses are abundant [15], and play a role in maintaining glycine stores in concert with VIAAT [32–34]. Functional roles of SLC7A10 in astrocytes, especially of the forebrain, are open to further research. In this regard, loss of function experiment would become a powerful tool and conditional knockout of the SLC7A10 gene in the Olig2 astrocytes is of particular interest and is an ongoing project for us.

## Conclusion

We demonstrated that Olig2-astrocytes in the lateral globus pallidus comprise a population that expresses neutral acid transporters, especially SLC7A10, at a high level.

Our findings are consistent with the recent discovery that SLC7A10 is expressed not only in neurons but also in a subset of astrocytes, and suggest that SLC7A10 exerts specific functions in Olig2-astrocytes of the adult brain.

## Methods

### Single-cell transcriptomic analysis for whole-brain cells

#### Initial clustering analysis

We analyzed the 2–3-month-old mouse brain scRNA-seq datasets (GSM3722100, GSM3722101, GSM3722102, GSM3722103, GSM3722104, GSM3722105, GSM3722106, GSM3722107) generated by Ximerakis et al. [12], using the Seurat v.3.0.0 R package [35]. We filtered out cells with more than 6000 or fewer than 250 detected genes, as well as those with higher than 30% of mitochondrial transcripts. We removed any genes that were detected in fewer than three cells. After quality control, we collected a total of 16,028 single cells with 14,658 genes. The datasets were log-normalized and variable genes were identified using the “FindVariableGenes” function. The eight datasets were integrated using canonical correlation analysis, “FindIntegrationAnchors” and “IntegrateData” functions [36]. Principal component analysis (PCA) was performed on the integrated datasets. Based on the top 30 principal components (PCs), clusters were identified with the “FindClusters” function using shared nearest-neighbor modularity optimization with clustering resolution set to 0.8. Dimensionality reduction was performed using “Uniform Manifold Approximation and Projection” (UMAP). We obtained 28 clusters and categorized them into 15 clusters of the major cell types, based on their transcriptional profiles (Additional file 1: Fig. S1). Genes that were differentially expressed in each cluster were identified using the “FindAllMarkers” function (Additional file 2: Table S1). The cell types were determined based on cell type-specific marker genes that were previously described [12, 37].

#### Second clustering analysis

Among the 15 clusters, we extracted a total of 3522 cells with 14,658 genes as an astrocyte (ASC) cluster (Fig. 1a, colored blue) and performed further classification of this cluster. The dataset was log-normalized, after which we identified variable genes and performed PCA. Finally, 11 groups (referred to as A1–A11) were identified in the ASC cluster based on the top 15 PCs, using the “FindClusters” function with resolution set to 0.8 (Fig. 2a). Genes that were differentially expressed in each group were identified using the “FindAllMarkers” function (Additional file 3: Table S2). Cells with gene expression level > 0 were defined as “gene-positive cells”, and those with gene expression level = 0 were defined as “gene-negative cells”. We classified a total of 3522 cells into Olig2(+)/GFAP(−) astrocytes (347 cells), Olig2(+)/GFAP(+) astrocytes (104 cells), Olig2(−)/GFAP(+) astrocytes (848 cells) and Olig2(−)/GFAP(−) astrocytes (2223 cells) (Fig. 1g).

#### Differentially expressed gene analysis

Genes that were significantly expressed in Olig2-astrocytes rather than GFAP-astrocytes were detected by applying the “FindMarkers” function within the MAST method [38] (Additional file 6: Table S5). Using DAVID 6.7 [39], GO analysis was performed for genes with an adjusted p value less than 0.05 (Additional file 5: Table S4).

### Animals and tamoxifen administration

We crossed Olig2 knock-in mice (Olig2^KICreER^) [1] with Gt (ROSA)26Sor^tm27.1(CAG-COP4*H134R/tdTomato)Hze^ reporter mice (Ai27 mice, Jackson Laboratory, USA, Stock No. 012567) (their progeny are hereafter termed Olig2^CreER^-Ai27 mice) for a laser microdissection experiment. In Ai27 mice, Cre-inducible channelrhodopsin (H134R) is fused with tdTomato, resulting in efficient and strong RFP fluorescence at the plasma membrane and clearly revealing cellular morphology without RFP immunostaining. Olig2^CreER^-Ai27 mice were maintained in a mixed genetic background (C57BL/6 and 129 S6/Sv strains). These mice were housed in standard cages with access to food and water ad libitum and controlled humidity (55%) and temperature (23 °C) under a 12-h light/dark cycle. All Olig2^CreER^-Ai27 mice (n = 8) used were male and 8–9 weeks old, and all protocols for the animal experiments were approved by the Animal Care Committee of Nara Medical University in accordance with the policies established in the NIH Guide for the Care and Use of Laboratory Animals. The study was carried out in compliance with the ARRIVE guidelines. The method for oral administration of tamoxifen was as previously described [3, 4]. Olig2^CreER^-Ai27 mice were allowed to access tamoxifen-containing chow ad libitum for 10 days. After tamoxifen administration, mice were housed for 3 weeks with normal feed to fully express fluorescent proteins. Feeding with tamoxifen-containing chow successfully enhanced recombination and subsequent tdTomato red fluorescence.

### Tissue preparation and immunohistochemistry for laser microdissection

Mouse brains were removed after cervical dislocation and immediately frozen in powdered dry ice. Coronal sections containing the LGP (Bregma − 0.58 to − 0.94) [40] were cut at 20-μm thickness on a cryostat (Leica CM1860, Leica Microsystems, Japan). Eight sections per mouse with 60-μm intervals were mounted on a PPS membrane slide (#11600294; Leica Microsystems). The slides were kept in the cryostat at – 20 °C for 1 h, placed on a hotplate at 40 °C for 35 s, and immediately dried under a hair dryer. Completely dried sections were fixed in ice-cold acetone for 4 min and again dried quickly. To identify GFAP-astrocytes, sections were incubated with GFAP antibody (1:60, MAB360, EMD Millipore, USA) diluted in PBS containing 10% BSA, 1% RNase inhibitor and 4% DTT for 5 min. After washing with PBS, they were incubated with Alexa 488-conjugated anti-mouse IgG (1:100, Jackson ImmunoResearch, USA). Blue-fluorescent Nissl stain (1:50, Neurotrace 435/455, Thermo Fisher Scientific, Japan) was used simultaneously as a counterstain.

We dissected out tdTomato-expressing cells with bushy morphology (Olig2-astrocytes) and Alexa 488-labeled cells (GFAP-astrocytes) from a single section of Olig2^CreER^-Ai27 mice using an LMD 6500 system (Leica Microsystems). For each astrocyte sample, about 500 cells were dissected from eight coronal sections per mouse and were subjected to RNA extraction.

### Tissue preparation and immunohistochemistry

After perfusion with 4% paraformaldehyde (PFA) in phosphate buffer, brains were removed and post-fixed in 4% PFA overnight (~ 12 h) at 4 °C. All brains were washed in PBS solution including 30% sucrose and snap-frozen; frozen sections of 30-µm thickness were then cut. All histological procedures were performed as described previously [3].

For primary antibody, anti-SLC7a10 (N-terminal) (1:200, rabbit polyclonal, GTX47874, GeneTex, USA) was used. Alexa 488-conjugated anti-rabbit IgG (1:1000, Jackson ImmunoResearch Laboratories, UK) was used for secondary antibody in immunofluorescence staining. Counterstaining was performed by blue-fluorescent Nissl stain (1:200, Neurotrace 435/455, Thermo Fisher Scientific). Fluorescence images were obtained with a confocal laser scanning microscope (Nikon C2-NiE, Japan). To obtain 3D deconvolution images, we used a confocal laser scanning microscope (Olympus FV3000, Japan). For immunoenzyme staining, we used anti-rabbit IgG combined with amino acid polymers and peroxidase (Histofine Simple Stain MAX PO Kit, 414141F, Nichirei Bioscience, Japan) for secondary antibody. The peroxidase color reaction was performed in diaminobenzidine tetrahydrochloride (DAB) solution (DAB Substrate Kit, SK-4100, Vector Laboratories, USA).

### RT-qPCR

Total RNA was isolated from each astrocyte type obtained from the LGP using a CellAmp Direct RNA Prep Kit (Cat. #3732, Takara, Japan). For cDNA synthesis, we used a PrimeScript RT reagent Kit (Cat. #RR037A, Takara). After pre-amplification using PreAmp SuperMix reagent (Cat. #95146-005, Quanta BioSciences, USA), RT-qPCR was performed using SYBR qPCR Mix (Cat. #QPS-201, Toyobo, Japan). The gene-specific primer sets are described in Additional file 8: Table S7.

### Statistics

Graphical data are presented as the mean ± SEM. Student’s t-test was used to compare mean values for unpaired data. Differences were considered significant when p < 0.05.

## Supplementary Information

**Additional file 1: Fig. S1.** Full-length gels corresponding to Fig. 2e. PCR analysis of selected transcripts in laser-microdissectedLGP (top), Olig2-astrocytes (middle) and GFAP-astrocytes (bottom). These samples derive from the same experiment and gels were processed in parallel.

**Additional file 2: Table S1.** Lists of significant DEGs (p < 0.01) in each cluster (related to Fig. 1).

**Additional file 3: Table S2.** Differential gene expression in astrocyte group (related to Fig. 2).

**Additional file 4: Table S3.** Cell number of Olig2-positive/GFAP-positive, Olig2-positive/GFAP-negative, Olig2-negative/GFAP-positive, Olig2-negative/GFAP-negative cells, in each group of Astrocytes.

**Additional file 5: Table S4.** Gene ontology (GO) terms (biological process) of A1 group by GO analysis (related to Fig. 2d).

**Additional file 6: Table S5.** 416 genes that were significantly expressed in Olig2-astrocytes rather than GFAP-astrocytes (p < 0.05, adjusted p value based on Bonferroni correction).

**Additional file 7: Table S6.** Solute carrier (SLC) genes extracted from the 416 genes whose expression was significantly biased to Olig2-astrocytes as compared to GFAP-astrocytes (Table S5) (related to Fig. 2e).

**Additional file 8: Table S7.** Primer sets for qPCR.

## Data Availability

All experimental protocols are described in the “Methods” section or the references therein. The data that support the findings of this study are available as Additional files.
