# Supplementary material for: Olig2-astrocytes express neutral amino acid transporter SLC7A10 (Asc-1) in the adult brain
Source: Mol Brain. 2021 Nov 8;14:163. doi: 10.1186/s13041-021-00874-8 (PMC8573876; doi:10.1186/s13041-021-00874-8)
Supplement: Supplementary file 1 — Additional file 1: Fig. S1. Full-length gels corresponding to Fig. 2e. PCR analysis of selected transcripts in laser-microdissectedLGP (top), Olig2-astrocytes (middle) and GFAP-astrocytes (bottom). These samples derive from the same experiment and gels were processed in parallel. [file 13041_2021_874_MOESM1_ESM.pdf]

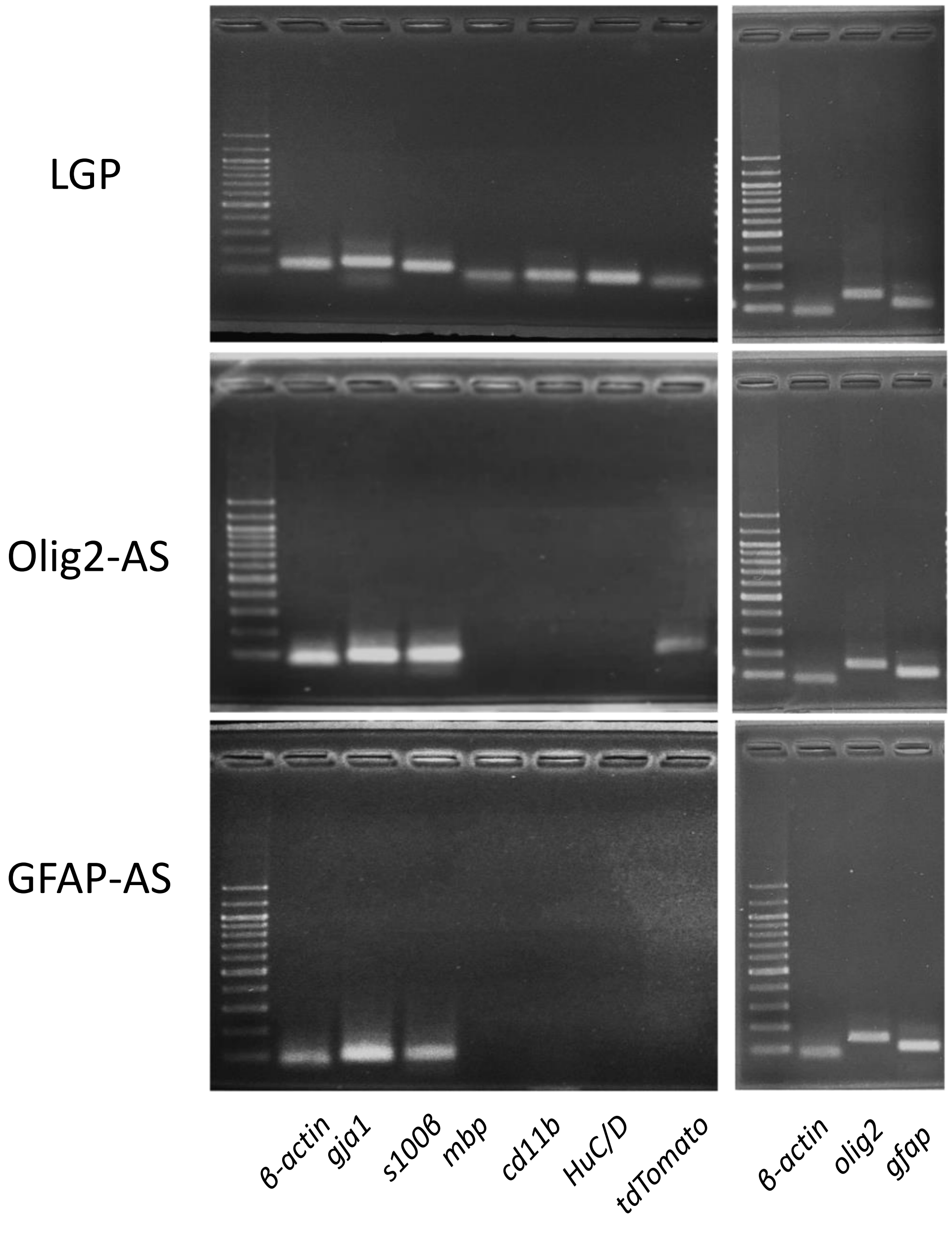

Additional file 1 : Fig. S1. Full-length gels corresponding to Fig. 2e.
PCR analysis of selected transcripts in laser-microdissected LGP (top), Olig2-astrocytes (middle) and GFAP-astrocytes (bottom) . These samples derive from the same experiment and gels were processed in parallel.
