# Supplementary material for: Olig2-astrocytes express neutral amino acid transporter SLC7A10 (Asc-1) in the adult brain
Source: Mol Brain. 2021 Nov 8;14:163. doi: 10.1186/s13041-021-00874-8 (PMC8573876; doi:10.1186/s13041-021-00874-8)
Supplement: Supplementary file 8 — Additional file 8: Table S7. Primer sets for qPCR. [file 13041_2021_874_MOESM8_ESM.docx]

Table S7

Primer sets for qPCR

| Target Gene | Forward (5'- 3') | Reverse (5'- 3') | Product length (bp) | NCBI reference |
| --- | --- | --- | --- | --- |
| *slc1a2* | ATGACAGCCACCTCAGCTCC | CACTGCTCCCAGGATGACAC | 122 | NM_001077514.4 |
| *slc1a3* | TGTGCTTCGGTTTCGTGATC | ACCAATCGCATGATGGCTTC | 94 | NM_148938.3 |
| *slc6a1* | TTCAGACTGTTGTCCTGTGC | AGCGGCCATGAACAAAATGC | 111 | NM_178703.3 |
| *slc6a9* | TGTTGGCGCTTTGTTTCTCC | TGACAGACGACAAAGCCATG | 142 | NM_001355175.2 |
| *slc6a13* | ACGTGTTCCAGCTCTTTGAC | TCCATAAACCCAAGCCACAC | 95 | NM_144512.3 |
| *slc7a10* | AAGCTGCTGGGCTACTTTTC | ATGAATCATGGCCAGGAAGC | 150 | NM_017394.4 |
| *olig2* | TGGGGGCTTGACAAAAGAAC | AACAAAGAGCTTCGCATCGC | 146 | NM_016967.2 |
| *gfap* | TCAATGCTGGCTTCAAGGAG | AGCGCCTTGTTTTGCTGTTC | 118 | NM_001131020.1 |
| *s100β* | TGCCCTCATTGATGTCTTCCA | GAGAGAGCTCGTTGTTGATAAGCT | 101 | NM_009115.3 |
| *gja1* | CTGCCGCAATTACAACAAGC | TTGGCATTTTGGCTGTCGTC | 144 | NM_010288.3 |
| *aldh1l1* | ATGAAAAGCTGTGCCCTGAG | TGCACAGCTTTGTTGAGGTC | 104 | NM_001356412.1 |
| *aqp4* | TTGAAGCCAGAGAGCCAAAC | AGGCCATGCTTGCACAATTC | 101 | NM_001308641.1 |
| *mbp* | AGAGGCTGGAAAGAAGAGAAGC | TTGTTCTGGATCGCATCTGC | 93 | NM_010777.3 |
| *cd11b* | ATTGCGGCAATGACAGCATC | TCCTCACCGTCATTTCTCAGAG | 130 | NM_001082960.1 |
| *huC/D* | AACAAGTGCAAGGGTTTCGG | TAAATGCTCAGGCCTTGTGC | 148 | XM_006510021.1 |
| *tdTomato* | ACATCCCCGATTACAAGAAGC | TTGTAGATCAGCGTGCCGTC | 130 | AY678269.1 |
| *beta-actin* | TTTGGCGCTTTTGACTCAGG | ACTTTGGGGGATGTTTGCTC | 80 | NM_007393.5 |
